# Effect of concentrated growth factor (CGF) on postoperative sequel of completely impacted lower third molar extraction: a randomized controlled clinical study

**DOI:** 10.1186/s12903-022-02408-7

**Published:** 2022-08-30

**Authors:** Sadam Ahmed Elayah, Xiang Liang, Karim Ahmed Sakran, Linyang Xie, Hamza Younis, Ahmed Es. Alajami, Junbo Tu, Sijia Na

**Affiliations:** 1 Key Laboratory of Shaanxi Province for Craniofacial Precision Medicine Research, College of Stomatology, Xi’an Jiaotong University, Xi’an, China; 2 Department of Oral and Maxillofacial Surgery, College of Stomatology, Xi’an Jiaotong University, Xi’an, Shaanxi 710004 China; 3 State Key Laboratory of Oral Diseases and National Clinical Research Center for Oral Diseases and Department of Oral and Maxillofacial Surgery, West China Hospital of Stomatology, Sichuan University, Chengdu, China; 4 Department of Oral and Maxillofacial Surgery, College of Dentistry, Ibb University, Ibb, Yemen

**Keywords:** Third molar, Impacted teeth, Postoperative complications, Platelet, CGF

## Abstract

**Background:**

The surgical extraction of impacted third molars is one of the most common procedures in oral and maxillofacial surgery, which associated with several postoperative complications. The aim of this clinical trial was to estimate the implication of concentrated growth factor (CGF) on postoperative sequelae after the completely impacted lower third molar extraction.

**Materials and methods:**

A total of 74 sides of 37 participants who had completely bilateral impacted lower third molars were enrolled in this split-mouth, randomized single‑blind, clinical trial. Surgical extraction was undertaken on both sides of the mandible. Randomization was achieved by opaque, sealed envelopes. The postoperative outcomes including wound healing, swelling and pain were clinically assessed at different-time intervals(1st, 3rd and 7th days). A p-value < 0.05 was considered statistically significant.

**Results:**

The wound healing index was significantly better in the test sides (P = 0.001). Regarding the facial swelling, the test sides had significantly less values than the control sides, particularly on the 1st (1.01 ± .57 vs. 1.55 ± .56) and 3rd days (1.42 ± 0.8 vs. 2.63 ± 1.2) postoperatively. Nonetheless, the swelling was disappeared within the 7th day in both sides. The pain scores of visual analog scale were no a statistically significant difference between both sides on the 1st day, meanwhile, the pain scores were significantly lower in the test sides compared with the control sides, especially on the 3rd (P = 0.001) and 7th days (P < 0.001) postoperatively.

**Conclusion:**

The application of CGF following the surgical extraction of lower third molar has accelerated the healing of soft tissues as well as reduced postoperative sequelae such as swelling and pain. Therefore, the CGF could be promoted among clinicians during the lower third molar surgical extraction.

*Trial registration*: This study was registered with the TCTR identification number TCTR20210325002 on 25/03/2021 at Thai Clinical Trials Register-Medical Research Foundation of Thailand (MRF). Also it was ethically approved from the institutional ethics committee at the Hospital of Stomatology, Xian Jiaotong University, Xian, China (No: 032), and has been conducted in accordance to the guidelines of the declaration of Helsinki. Written informed consent was obtained from all participants in the study.

**Supplementary Information:**

The online version contains supplementary material available at 10.1186/s12903-022-02408-7.

## Background

Third molar extraction is amongst the most frequent procedures done in a routine dental clinic [1]. However, many various types of postoperative sequelae have been observed due to acute inflammatory response to surgical trauma [2]. Reduction of these sequelae becomes crucial for the success of surgical procedures [3, 4]. Prolonged periods of pain and inflammation are mediated by release of local prostaglandins. Postoperative swelling emerges as a result of tissue damage during surgical extraction, the raising of muscular attachments and as a consequence of direct damage to blood and lymph vessels [5]. On other hand, surgical extraction of an impacted third molar routinely demands massive bone removal in order to expose the impacted tooth, maintenance of the alveolar process and distally periodontal tissue to second molars is a critical prerequisite after surgery [6]. Upon literature study, it was noted that postoperative discomfort is highly prevalent following surgery of impacted third molars [7–10]. Hence, several therapeutic approaches have been implemented to minimise the incidence of postoperative sequelae [11] such as local application of PRF [12], using of Aprotinin [13], using of Ibuprofen and Methylprednisolone [14], Administration of Dexamethasone [15], using of a tube drain [3], using of laser therapy [16], and using piezoelectric bone surgery [17]. Even though all these approaches in management of postoperative complications, adverse effects still pose a major challenge [18, 19]. In recent years, the healing of the dental socket after tooth extraction has been emerged as a topic for debate in advanced dentistry. Regenerative medicine is one of the most key tasks of today's rehabilitation therapies, and it is one of the most difficult to achieve.

Numerous studies of growth factors concluded that the best tissue regenerative stimulus are the autologous growth factors, which have been clinically proven to promote tissue regeneration [8]. Growth factors are bioactive proteins which control the process of bone and soft tissues regeneration [8]. Platelet is one of the major resources of autogenous growth factors [7]. Various platelet concentrates such as platelet-rich plasma (PRP), platelet-rich fibrin (PRF), and concentrated growth factor (CGF); it is a novel concentrated platelet substance that was established by Sacco in 2006 [20] and is being considered as a new kind of biological scaffold which is rich in platelets and fibrin. CGF plays a significant role in that it includes numerous types of growth factors and fibrin used in wound healing [9].

The majority of current studies are focused on the efficacy of CGF applications in dentistry [21]. Its application has been recommended for a variety of situations, including filling postextraction [22].

A comprehensive review of CGF represents significance in the concept of personalised therapy. However, there is a limitation of scientific evidence about the utilisation of CGF at this time. It is necessary to undertake further scientific and clinical experiments in order to better understand the properties and clinical applicability of CGF [23–25]. Therefore, the purpose of this randomized clinical trial study was to determine whether locally application of CGF in the dental socket could substantially enhance wound healing and minimise postoperative sequelae in patients who underwent surgical extraction of completely impacted lower third molars. The question, does local application of CGF in the dental socket significantly minimize postoperative sequel in patients who have been extracted completely impacted lower third molar?

## Materials and methods

A split-mouth, randomized single -blind, clinical trial study was conducted on 37 participants (74 sides) who came to the stomatology hospital of Xian Jiaotong University and had underwent surgical extraction of completely, bilaterally, and symmetrically impacted lower third molars during the period from Apr 2021 to Dec 2021. This study was registered with the TCTR identification number TCTR20210325002 on 25/03 /2021 at Thai Clinical Trials Register-Medical Research Foundation of Thailand (MRF).Also it was ethically approved from the institutional ethics committee at the Hospital of Stomatology, Xian Jiaotong University, Xian, China (No: 032), and has been conducted in accordance to the guidelines of the declaration of Helsinki. Written informed consent was obtained from all participants in the study.

### Selection and preparation of participants

All the participants have undergone physical and radiographic examination preoperatively. They have been selected based on the following criteria: (1) Had symmetrically, bilaterally, horizontal or vertical completely impacted lower third molars with comparable hardness grade that was assessed based on Pederson's description [26] using panoramic radiographs (the difficulty index ranged from 7 to 10), (2) Age ranged from 18 to 38 years, (3) Good oral hygiene, (4) Cooperative patients who able to complete the follow up appointments, (5) No previous history of systemic diseases, (6) No periodontal diseases, especially in the work area, (7) No history of long term steroid therapy, and (8) No history of radiotherapy in the head and neck region. All the patients were informed about the treatment protocol and the purpose of the study, and patients had undergone surgical extraction of bilateral impacted lower third molars in a single appointment [27].

### Randomization

The participants were evaluated by outcome assessors (S.E, L.X & H.Y). Before the completely, bilaterally-impacted mandibular third molar surgery, opaque, sealed envelope [28] was utilised to randomly pick the side wherein CGF was to be put, selected by the patient. The envelopes contained cards labelled ‘R’ or ‘L’, which indicated the surgical side to receive CGF application. The envelopes were opened by the surgeon (N.S) after the patients made their selection. There were two sides: A test side, which received CGF treatment after the tooth extraction and a control side, which did not receive CGF treatment.

Outcome assessors were not aware of the CGF side. Therefore, this study was (assessor‑blind) single‑blind clinical trial [28].

### Study variables

The primary variable of this clinical study was CGF application.

The primary outcome variables were soft tissue healing around the extraction socket and swelling.

The secondary outcome variables was pain.

### CGF preparation

**Fig. 1:**
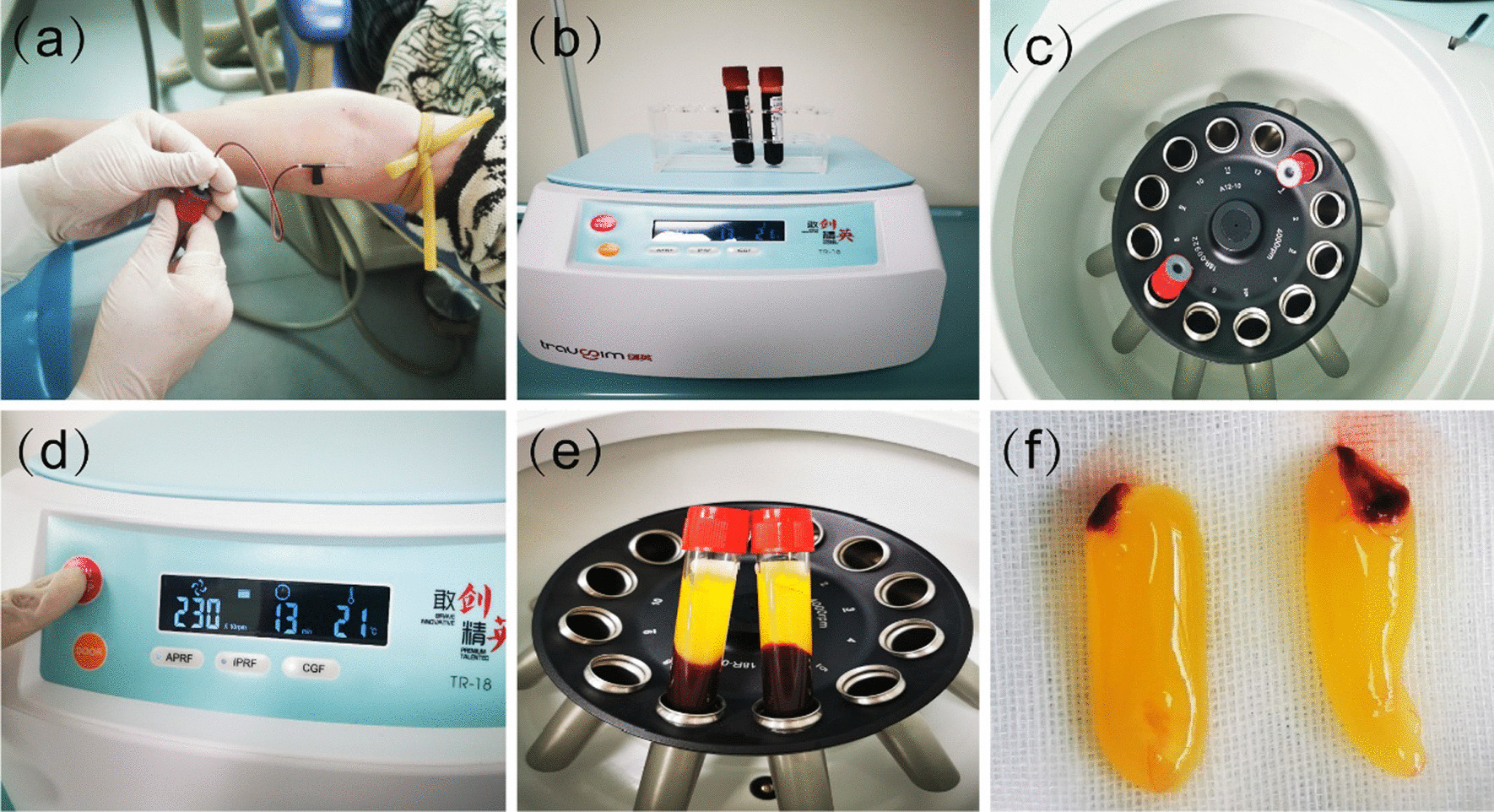
Preparation of CGF (**a**) blood withdraw, (**b**) sterilised 10 ml tubes, (**c**, **d**) CGF centrifuge equipment, trausim, (**e**) after centrifugation, (**f**) CGF fibrin gel

The samples of autologous CGF were obtained from patients' freshly venous blood. They were divided into two sterilised 10 ml tubes without anticoagulants and centrifuged immediately [20] using a CGF centrifuge equipment (trausim, DL4015, Dental Regenerative Centrifuge, China) under the following measures: speed, 230*10 rpm; running time, 13 min; chamber temperature, 21 (Fig. 1a–f).

### Sample size calculation

Sample size was calculated with the G*power 3.0.10 software. The minimum sample size required was 31 subjects per group. This showed that 37 subjects (37 test side and 37 control side) would be sufficient to obtain 90% power in detecting a statistical difference between the test and control sides, with a target significance level of 0.05.

In addition, it was carried out based on previous similar studies [12, 29–31].

### Surgical protocol

Before surgery, patients rinsed their mouths with 0.12% clorhexidine gluconate as an antiseptic mouthwash for 60 s. All the operations were done by the same experienced oral surgeon, who followed the same protocol (Additional file 1: Fig. S1 a, b, c, d) as following steps; Modified Ward’s Incision [32] was applied under local anaesthetic, 2% lidocaine with 1:80,000 epinephrine (1/200,000) (Additional file 1: Fig. S1 b). After bilaterally extraction, the sockets were irrigated using 60 mL of sterile saline. CGF fibrin was placed in one socket (test side), which was randomly selected using an opaque, sealed envelope. The opposite socket left to heal naturally (control side). The flaps were sutured with 4/0 atraumatic silk sutures (1/2 cutting edge, 75 cm) (Additional file 1: Fig. S1d). Postoperatively, amoxicillin (500 mg/8 h for five days), 0.2% chlorhexidine mouthwash (twice per day for seven days) and paracetamol (500 mg, every 4–6 h) were prescribed by the surgeon. Providing postoperative instructions for all patients. After one week, sutures were removed.

### Evaluation of outcomes

The wound was carefully irrigated with saline. The postoperative outcomes were clinically evaluated in all the enrolled cases and at different time intervals. On 7th day, the wound healing was assessed using Landry et al. index, which assigns a score from 1 to 5, 1 indicates “very poor healing” while 5 indicates “excellent healing” [3, 33].

The assessment of the facial swelling was done with the use of a horizontal and vertical guidance that included four reference points: the outer canthus of the eye, the mandibular angle, the attachment of the ear lobe, and the corner of the mouth. The postoperative facial swelling was evaluated on the 1st, 3rd, and 7th days after the surgery and was determined by taking the arithmetic mean of the two measurements. This was calculated by observing the difference between measurements taken before and after dental extraction and dividing the value by the value obtained before dental extraction, then multiplying it by 100 to obtain the percentage of facial swelling as presented in a previous study [3].

The visual analog scale (VAS) has proven to be a reliable and sensitive approach for pain recording after surgical intervention, and it is now frequently used in clinical practice [34–36]. Thus, the postoperative pain was measured on the 1st, 3rd, and 7th days after the surgery using the VAS, which ranges between 0 (no pain) and 10 (worst pain) [34].

### Statistical analysis

SPSS version 18 (Chicago, USA) was used to calculate all the statistical analysis. The descriptive data were expressed as mean (SD) and median (Min–Max) or as frequency and percentage where appropriate. The Chi-square test was performed to analyse the difference in wound healing index and VAS between the two sides. Mann–Whitney test was used to compare the facial swelling measure between the sides. The significance level was set at 0.05.

## Results

A total of 74sides of 37 participants (19 males and 18 females) with an average age of 25 years were enrolled in this prospective clinical trial study. Regarding the wound healing index, 56.8% of cases in the control side showed good index while 73% of cases in the test side had very good index. In this context, the two sides were compared favourably with better wound healing in the test side (P = 0.001) (Table 1). In term of facial swelling, the test side had significantly less swelling than the control side, particularly on the 1st day (1.01 ± 0.57 vs. 1.55 ± 0.56) and 3rd day (1.42 ± 0.8 vs. 2.63 ± 1.2) postoperatively. Nonetheless, the swelling was disappeared within the postoperative 7th day in both sides (Table 2).

**Table 1:** Comparison of wound healing index between the test and control sides after the 7th day using the Chi-square test

Wound healing index	Control side n (%)	Test side n (%)	*P*
Very poor (1)	1	0	.001
Poor (2)	5	0	
Good (3)	21	10	
Very Good (4)	10	27	
Excellent (5)	0	0

**Table 2:** Comparison of swelling between the test and control sides during the different time intervals using Mann–Whitney test

Swelling	24 h		3rd day		7th day	
	Mean (SD)	Median (Max–Min)	Mean (SD)	Median (Max–Min)	Mean (SD)	Median (Max–Min)
Test side	1.01 (0.57)	1 (0.5–2.5)	1.58 (0.61)	1.5 (0.3–3)	1.23 (1.25)	1 (0.0–4)
Control side	1.55 (0.563)	1.5 (0.5–3)	1.05 (0.59)	1 (0.5–0.25)	0.69 (0.79)	0.5 (0.0–2.5)
P-value	.001		.001		.084	

Although there was no statistically significant difference in the VAS pain scores between the sides on the first postoperative day, the test side had shown significant reduction in the pain scores during the third and seventh days as compared with the control one (P = 0.001 and P < 0.001 postoperatively, Table 3).

**Table 3:** Comparison of pain scores on visual analog scale between the test and control sides during the different time intervals using the Chi-square test

Visual analog scale (VAS)	24 h		3rd day		7th day	
	Test.s	Cont. s	Test.s	Cont. s	Test s	Cont. s
No pain	0	0	6	0	34	16
Mild pain	16	11	24	15	3	18
Moderate	14	13	7	17	0	2
Severe	7	13	0	4	0	0
Very Severe	0	0	0	1	0	1
Worst pain	0	0	0	0	0	0
P-value	.251		.001		.000	

The treatment was well accepted by all participants, and there were no serious adverse effects such as alveolitis, infection, paraesthesia, fracture, etc.

## Discussion

The question of this clinical trial was “does local application of CGF in the dental socket significantly minimize postoperative sequel in patients who have been extracted the completely impacted lower third molar?.”

According to the current study's findings, (CGF) is a novel autogenous therapy, which has been reduced the postoperative sequel associated with third molar surgical extraction.

In term of wound healing index, our findings showed statistically significant differences (P = 0.001) between both sides, with better outcome in the test sides compared to the control sides. These findings were consistent with the findings of other previous studies [8, 37]. Similarly, Fiorillo et al. [22] concluded that CGF is an effective aid in accelerating the processes of soft-tissue regeneration. The CGF helps to improve wound stability, which is crucial to the creation of a new connective tissue attachment to the root surface [8]. The CGF is an efficient surgical haemostatic substance that also stimulates epithelial, endothelial, and epidermal regeneration while also minimising dermal scarring. Because of the large concentration of leukocytes, CGF exhibits some antibacterial properties. Hence, it has anti-angiogenic properties and may be used to treat chronic non-healing wounds [37]. Srinivas et al. reported that in post-extraction sockets, the wound healing index was better in the PRF group than in the control group [12].

Regarding the facial swelling, The use of PRF had no significant influence on the severity of facial swelling after the surgical extraction of teeth as reported in a previous study [38]. By contrast, Ozgul et al. [39] found that applying the PRF following wisdom teeth extraction had significantly diminished the facial swelling on the postoperative 1st and 3rd days. However, no statistically significant differences were noticed between the test and control sides on the postoperative 7th day. On the other hand, Koyuncu et al. [40] reported a statistically significant difference in postoperative swelling between the CGF group and control group on the 3rd and 7th days after partially third molar extraction. In this context, the current findings had showed statistically significant difference in the facial swelling between the test and control sides on the 1st and 3rd days postoperatively. Nonetheless, the swelling was disappeared within the 7th day in the two sides.

In this current study, the pain scores of visual analog scale (VAS) were no a statistically significant difference between both sides on the 1st day. Meanwhile, the pain scores were significantly lower in the test sides compared with the control sides on the 3rd (P = 0.001) and 7th days (P < 0.001) postoperatively. These findings were quite comparable to findings of Koyuncu et al. explored the role of CGF on postoperative pain and found that the severity of postoperative pain in the CGF group was lower than the control group, and that there was a statistically significant difference between the both groups for the first 7 days [40]. Qiao [41] and Qin [42] discovered that concentrated growth factor includes more growth factors than other platelet-based products. Thus, the CGF as contrast to PRP, does not disintegrate immediately after the application. This justifies our finding on the 1st day.

Bilginaylar et al. [17] have been compared between four groups (1-Traditional osteotomies with hand-piece burs group, 2-Traditional osteotomies and platelet-rich fibrin (PRF), 3-Osteotomies with piezoelectric group, and 4-Osteotomies with piezoelectric and PRF), who reported that there was no significant difference in pain on days 1, 3, 5, and 7, swelling and trismus between the control and other groups. Similarly, Sivolella et al. [43] and Barone et al. [34] who reported that no significant difference in pain on days 1, 3, 5, and 7 between the piezo-surgery and control groups. Barone et al. [34] also reported that no significant difference swelling on days 1, 3, and 7. The local application of PRF to the dental socket after lower wisdom teeth extraction helps to reduce pain and swelling [12].

Some recent systematic reviews had found that platelets have the potential to play a critical role in tissue regeneration because they are repositories of growth factors, which are essential for the regenerative procedures [21, 24]. The consistency of the CGF is the most important feature, which allows it to act as a growth factor repositories and natural scaffolding. Basically, the CGF is an improved version of the PRF, with a firmer fibrin matrix and high amounts of cytokines and growth factors [24].

The platelet containing preparations (including CGF) derived from human blood contain many growth factors such as bone morphogenetic protein (BMP), platelet-derived growth factor (PDGF), insulin-like growth factor (IGF), vascular endothelial growth factor (VEGF), transforming growth factor-β1 (TGF-β1), and transforming growth factor-β2 (TGF-β2), which also play a key role in bone healing [44, 45]. These growth factors attract the undifferentiated mesenchymal cells to the wound site, thus facilitating angiogenesis, chemotaxis, and cell proliferation [46]. Neither the U-CGF group nor the C-CGF group showed any significant inflammatory reactions or scarring. Thus, CGF may be used therapeutically to stimulate tissue regeneration either alone or in combination with other biomaterials [47].

In the area of dentistry, (CGF) has several applications, including the dentin–pulp complex regeneration [48], defects of periodontium [37], alveolar osteitis treatment [27], ridge augmentation surgeries, recession coverage, sinus lift, cystectomy, mixed with autologous bone graft and also used as a membrane during dental implantation, etc [21, 24].

The present findings should be taken in the context of its limitations. One of the limitations was that patients had undergone bilateral surgical extraction at the same appointment, hence they might not be able to accurately distinguish the level of pain on each side, especially on the 1^st^ day. Also, small sample size.In addition, the present trial focused mainly on clinical and short-term outcomes. Therefore, these limitations should be considered in further studies in order to confirm these preliminary findings.

## Conclusion

This clinical study shows that the local application of CGF had a significant effect on postoperative complications such as delayed wound healing, swelling and pain after completely impacted lower third molar surgical extraction. Thus, The CGF could be promoted among clinicians during the surgical extraction. Especially, the CGF prepration procedures are simple and economic.

## Supplementary Information

**Additional file 1: Fig. S1.** Lower third molar extraction (a) Impacted lower third molar ( b) Modified Ward’s Incision (c) Tooth separation after bone removal (d) Suturing.

## Data Availability

The datasets used and/or analysed during the study are available from the corresponding author on reasonable request.

## Abbreviations

CGF: Concentrated growth factors
PRF: Platelet rich fibrin
PRP: Platelet rich plasma
VAS: Visual analog scale
