# Supplementary material for: Effect of concentrated growth factor (CGF) on postoperative sequel of completely impacted lower third molar extraction: a randomized controlled clinical study
Source: BMC Oral Health. 2022 Aug 30;22:368. doi: 10.1186/s12903-022-02408-7 (PMC9426240; doi:10.1186/s12903-022-02408-7)

**Figure S**1 ; Lower third molar extraction (a) Impacted lower third molar ( b) Modified Ward’s Incision (c) Tooth separation after bone removal (d) Suturing.

c. Tooth separation after bone removal d. Suturing

a. Impacted lower third molar b. Modified Ward’s Incision


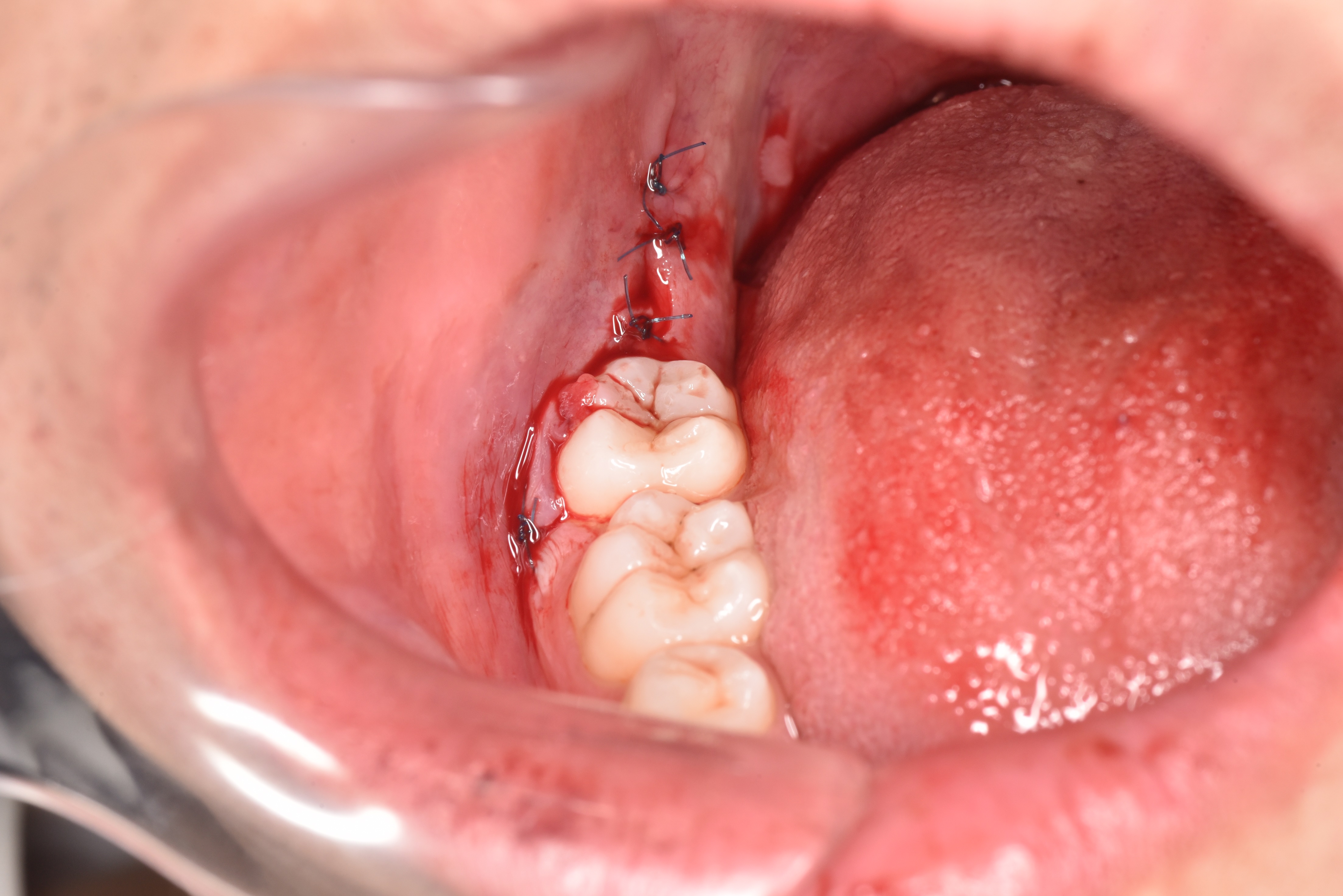


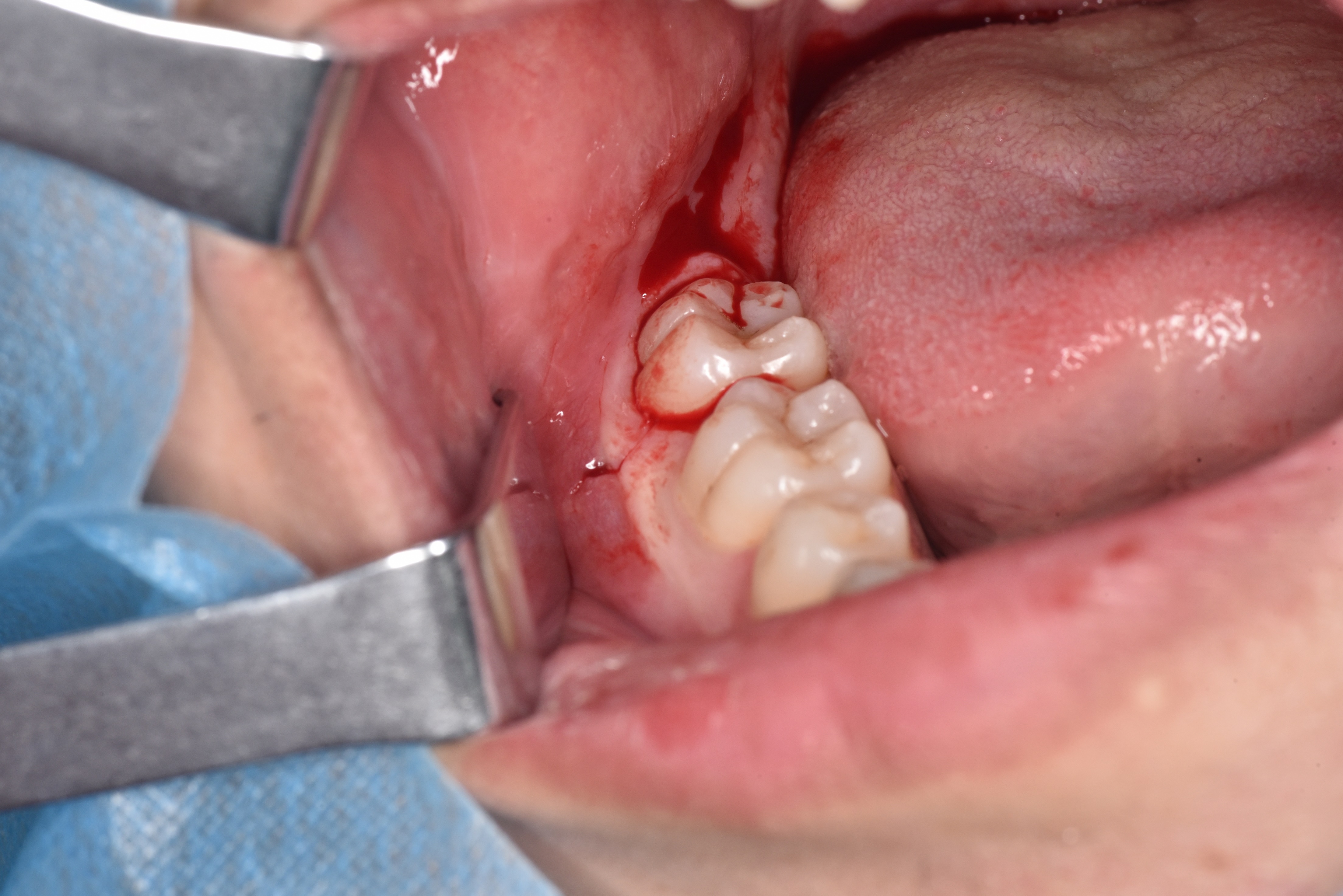


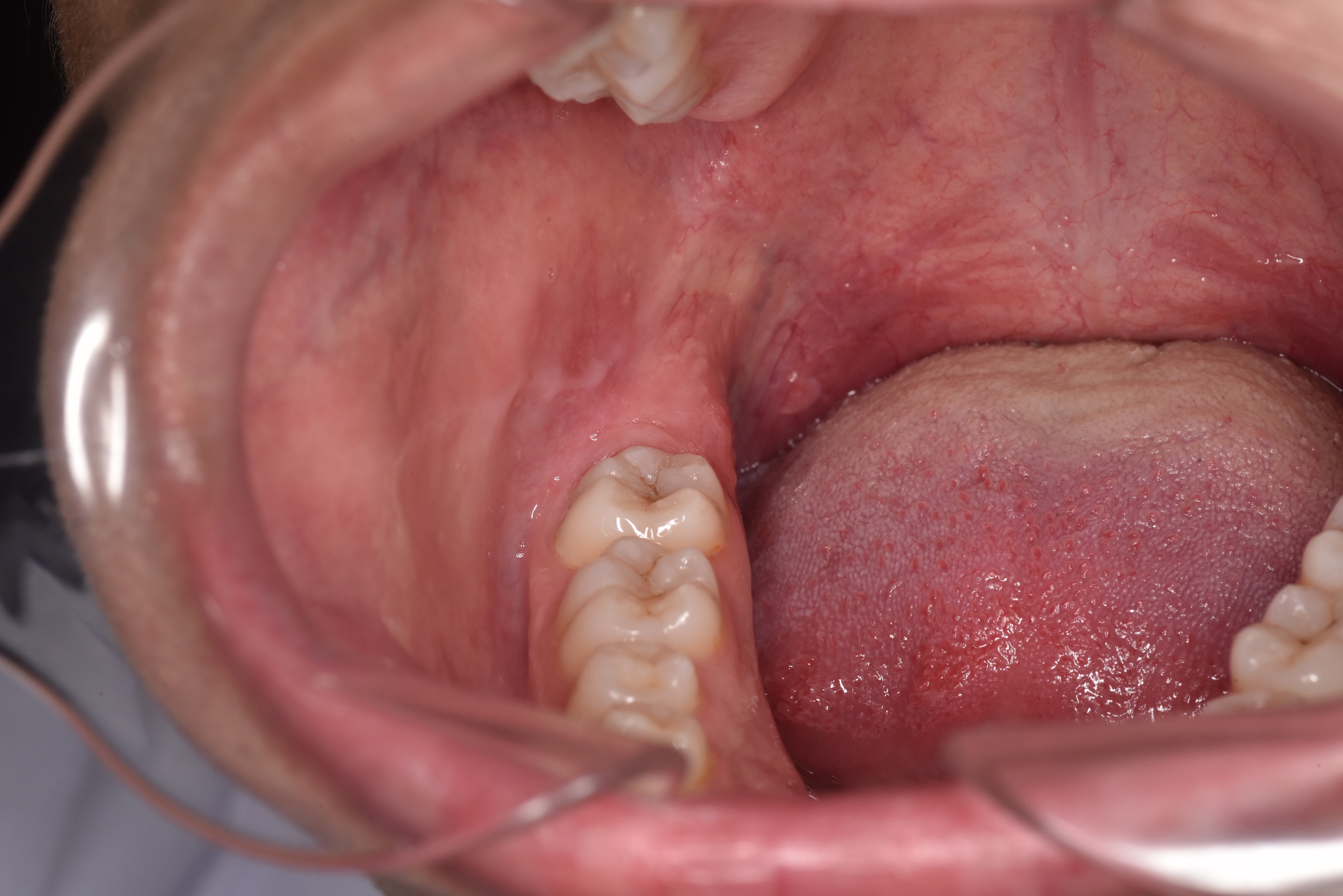


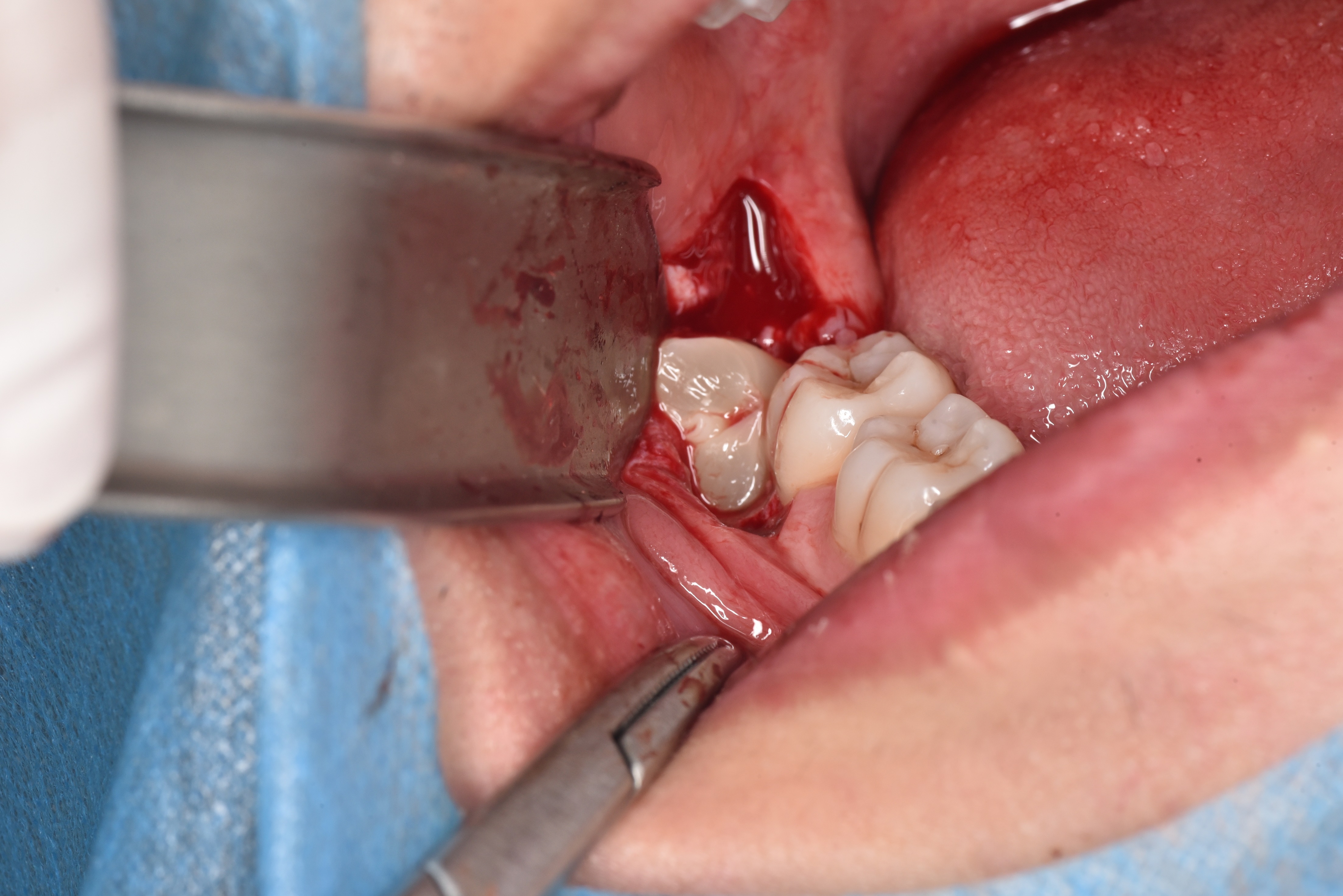

Supplement: Supplementary file 1 — Additional file 1: Fig. S1. Lower third molar extraction (a) Impacted lower third molar ( b) Modified Ward’s Incision (c) Tooth separation after bone removal (d) Suturing. [file 12903_2022_2408_MOESM1_ESM.docx]
